# Child Maltreatment History, Deployment-Related Traumatic Events, and Past 12-Month Cannabis Use Among Veterans in Canada: Antécédents de maltraitance d'enfants, événements traumatisants liés au déploiement, et utilisation du cannabis dans les 12 derniers mois chez les anciens combattants du Canada

**DOI:** 10.1177/07067437231192740

**Published:** 2023-08-11

**Authors:** Tracie O. Afifi, Tamara Taillieu, Samantha Salmon, Ashley Stewart-Tufescu, Jitender Sareen, Murray W. Enns, Natalie Mota, Shay-Lee Bolton, R. Nicholas Carleton, Alexandra Heber, Linda VanTil

**Affiliations:** 1 Departments of Community Health Sciences and Psychiatry, 8664 University of Manitoba, Winnipeg, MN, Canada; 2 Department of Community Health Sciences, 8664 University of Manitoba, Winnipeg, MN, Canada; 3 Faculty of Social Work, 8664 University of Manitoba, Winnipeg, MN, Canada; 4 Department of Psychiatry, 8664 University of Manitoba, Winnipeg, MN, Canada; 5 Department of Clinical Health Psychology, 8664 University of Manitoba, Winnipeg, MN, Canada; 6 Department of Psychology, University of Regina, Regina, SK, Canada; 7 Department of Psychiatry and Behavioural Neurosciences, 3710 McMaster University, Hamilton, ON, Canada; 8 26621 Veterans Affairs Canada, Charlottetown, PE, Canada

**Keywords:** veterans, cannabis, marijuana, hashish, substance use, child abuse, neglect, child maltreatment, mental disorders, chronic pain.

## Abstract

**Objective:**

Cannabis use among veterans in Canada is an understudied public health priority. The current study examined cannabis use prevalence and the relationships between child maltreatment histories and deployment-related traumatic events (DRTEs) with past 12-month cannabis use including sex differences among Canadian veterans.

**Method:**

Data were drawn from the 2018 Canadian Armed Forces Members and Veterans Mental Health Follow-up Survey (response rate 68.7%; veterans only *n* = 1,992). Five child maltreatment types and 9 types of DRTEs were assessed in relation to the past 12-month cannabis use.

**Results:**

The prevalence of lifetime and past 12-month cannabis use was 49.4% and 16.7%, respectively. Females were less likely than males to report lifetime cannabis use (41.9% vs. 50.4%; odds ratio [OR] 0.71; 95% CI, – 0.59 to 0.86). No sex differences were noted for past 12-month cannabis use (14.1% vs. 17.0%; OR 0.80; 95% CI, 0.60 to 1.07). Physical abuse, sexual abuse, neglect, any child maltreatment, most individual DRTEs, and any DRTE were associated with increased odds of past 12-month cannabis use after adjusting for sociodemographic and military variables. Some models were attenuated and/or nonsignificant after further adjustments for mental disorders and chronic pain conditions. Sex did not statistically significantly moderate these relationships. Cumulative effects of having experienced both child maltreatment and DRTEs compared to DRTEs alone increased the odds of past 12-month cannabis use. Statistically significant interaction effects between child maltreatment history and DRTE on cannabis use were not found.

**Conclusions:**

Child maltreatment histories and DRTEs increased the likelihood of past 12-month cannabis use among Canadian veterans. A history of child maltreatment, compared to DRTEs, indicated a more robust relationship. Understanding the links between child maltreatment, DRTEs, and cannabis use along with mental disorders and chronic pain conditions is important for developing interventions and improving health outcomes among veterans.

## Abrégé

**Objectif:**

L’utilisation du cannabis chez les anciens combattants du Canada est une priorité de santé publique sous-étudiée. La présente étude a examiné la prévalence de l’utilisation du cannabis et les relations entre les antécédents de la maltraitance d’enfants et les événements traumatisants liés au déploiement (ETLD) l’utilisation de cannabis des 12 derniers mois y compris les différences entre les sexes parmi les anciens combattants canadiens.

**Méthode:**

Les données ont été tirées de l’Enquête de suivi 2018 sur la santé mentale des membres des Forces armées canadiennes et des vétérans (ESSMRFC, taux de réponse 68,7 %; vétérans seulement n = 1 992). Cinq types de maltraitance d’enfants et 9 types d’ETLD ont été évalués en relation aux 12 mois passés d’utilisation du cannabis,

**Résultats:**

La prévalence de durée de vie et des 12 derniers mois de l’utilisation du cannabis était de 49,4 % et 16,7 %, respectivement. Les femmes étaient moins susceptibles que les hommes de déclarer une utilisation de durée de vie du cannabis (41,9 % c. 50,4 %; rapport de cotes 0,71, IC à 95 % – 0,59 à 0,86). Aucune différence entre les sexes n’a été notée dans les 12 derniers mois d’utilisation du cannabis (14,1 % c. 17,0 %; rapport de cotes 0,80, IC à 95 % = 0,60 à 1,07). Abus physique, abus sexuel, toute maltraitance d’enfants, la plupart des ETLD individuels, et tout ETLD étaient associés à des probabilités accrues d’utilisation du cannabis des 12 derniers mois après ajustement pour les variables sociodémographiques et militaires. Certains modèles ont été atténués et/ou non significatifs après d’autres ajustements pour les troubles mentaux et les affections de douleur chronique. Le sexe ne modérait pas statistiquement et significativement ces relations. Les effets cumulatifs d’avoir vécu à la fois la maltraitance d’enfants et les ETLD comparés aux ETLD seuls augmentaient les probabilités d’utilisation du cannabis des 12 derniers mois. Les effets d’interaction statistiquement significatifs entre les antécédents de maltraitance d’enfants et les ETLD sur l’utilisation du cannabis n’ont pas été observés.

**Conclusions:**

Les antécédents de maltraitance d’enfants et les ETLD augmentaient la probabilité d’utilisation de cannabis au cours des 12 derniers mois chez les anciens combattants canadiens. Des antécédents de maltraitance d’enfants, comparés aux ETLD, indiquaient une relation plus robuste. Comprendre les liens entre la maltraitance d’enfants, les ETLD et l’utilisation du cannabis avec les troubles mentaux et les conditions de douleur chronique est important pour développer des interventions et améliorer les résultats de santé chez les anciens combattants.

## Introduction

Mental health disorders and substance use are public health concerns in military and veteran populations worldwide. In 2002, the Canadian Community Health Survey Cycle 1.2, Canadian Forces Supplement (CCHS-CFS) was conducted and provided the first nationally representative mental health snapshot of the Canadian Armed Forces (CAF).^1,2^ Data indicated that 14.9% of CAF personnel met the criteria for a past 12-month mental disorder and 34.4% had heavy alcohol use.^1^ In 2013, the Canadian Forces Mental Health Survey provided an update regarding the mental health of the CAF and found the prevalence of past 12-month mental disorders and alcohol use disorder was 16.5% and 4.5%, respectively.^3,4^ The prevalence of self-reported past 12-month mental disorders among Canadian veterans ranges from 6.2% to 9.2%.^5^ Importantly, cannabis use was not assessed in these previous Canadian studies. Cannabis use among veterans remains an understudied public health priority requiring more research to better clarify risks and benefits in this population.^6^ A recent review of the literature on cannabis use among veterans indicated that although causal relationships cannot be determined with the current evidence, potential harms related to cannabis may include increased likelihood of using other substances, mental disorders, and self-harm or suicidality.^7^ Currently, there is limited knowledge specifically on cannabis use among veterans in Canada.

What is known from a nationally representative United States (US) sample from 2015 to 2017 is that past 12-month cannabis use ranged from 6% to 34% among veterans depending on age group and sex and was lower than past 12-month alcohol use (64% to 91%).^8^ Data from another nationally representative US veteran sample indicated that the lifetime and past 12-month prevalence of cannabis use among US veterans was 32.5% and 7.3%, respectively.^9^ A recent review on cannabis use among veterans found that 93% of the available research involves US veterans.^7^ The absence of similar Canadian data may be inhibiting effective Canadian health-care provision. Notably, Veteran Affairs Canada (VAC) reimbursements for medically authorized cannabis costs for 2019–2020 were $85 million covering 10 million grams of cannabis, which has increased substantially since 2011.^10^ Preliminary Canadian veteran data indicates that reimbursements from VAC for cannabis use for medical purposes are related to mental and physical health conditions, chronic pain, poor functioning, distress, suicidal ideation, problems with finances, and problems with family.^11^ Importantly, the US Department of Veterans Affairs health-care providers do not recommend the use of cannabis nor will Veterans Affairs pay for medical cannabis prescriptions from any source,^12^ which further highlights the need for more Canadian research.

Understanding how trauma may be related to cannabis use among veterans is another important knowledge gap. From a general population perspective, we know that child maltreatment is related to poor mental health and substance use.^13–17^ Military personnel can be exposed to DRTEs during service, which may have an impact on their mental health and substance use.^1,18^ More specifically, exposure to combat and/or peacekeeping on deployment has been associated with mental disorders among both serving men and women.^2^ Nearly half (47%) of CAF Regular Force personnel had a history of child abuse, as compared to 33% of the general population, and a child abuse history and DRTEs were both individually and cumulatively associated with increased odds of suicidal behaviours^19^ and mental disorders.^20^ Importantly, child abuse history has also been associated with alcohol use disorder among serving CAF personnel.^4^ From a theoretical perspective, cannabis might be used to self-medicate as a means of coping with child maltreatment histories and DRTEs. Further examination of these relationships may inform prevention efforts and provide clinical insights to increase knowledge about and reduce the potentially harmful use of cannabis among veterans. Although, it is necessary to note that veterans may use cannabis for reasons other than coping with child maltreatment or DRTEs.

More work is also needed to better understand possible sex differences related to cannabis use among veterans. More specifically, knowledge gaps related to sex and cannabis use are problematic because substance use may not be the same for male and female veterans.^8^ There are results from older US data wherein female veterans may have more substance misuse compared to male veterans,^21^ but newer data specific to cannabis indicates that female veterans are less likely to use cannabis than male veterans.^9,22^ As well, recent data among female US veterans indicates that 11% regularly used cannabis and cannabis use was related to alcohol use, posttraumatic stress disorder (PTSD) symptoms, childhood trauma, and sexual trauma.^23^

Mental disorder and chronic pain may be important to consider when examining trauma and cannabis use. Past 12-month cannabis use was associated with increased odds of alcohol use disorder, opioid use disorder, drug use disorder, tobacco use disorder, any mood disorder, and any anxiety disorder among a representative sample of US veterans.^9,24–26^ Similarly, other research using a representative US veteran sample found a statistically significant relationship between cannabis use and mental disorders among veterans with subthreshold/full PTSD.^25^ Medical use of cannabis is also common among military and veteran populations for the management of PTSD symptoms and chronic pain.^27^ More frequent cannabis use was found to be more likely among US veterans with chronic pain and cannabis use disorder was more likely among those reporting recent pain.^28^

The current study objectives are as follows. First, to compute the prevalence of lifetime and past 12-month cannabis use. Second, to examine if veterans with (a) a history of child maltreatment and (b) DRTEs are more likely to use cannabis in the past 12 months after adjusting for sociodemographic variables, military variables, mental disorders, and chronic pain conditions, and whether these relationships differ by sex. Third, to determine whether a cumulative or interaction effect for child maltreatment history and DRTEs exists in relation to the past 12-month cannabis use.

## Method

### Data and Sample

Statistics Canada conducted the CCHS-CFS in 2002, which included a representative sample of 5,155 active duty Regular Forces CAF personnel.^29^ In 2018, the Canadian Armed Forces Members and Veterans Mental Health Follow-up Survey (CAFVMHS) data were collected as a follow-up survey to the 2002 CCHS-CFS. Statistics Canada reinterviewed 68.7% (*n* = 2,941 including *n* = 949 actively serving Regular Force CAF personnel and *n* = 1,992 veterans) of those eligible.^30^ The current study only included veterans because cannabis use was only asked among veterans. More details related to the CAFVMHS have been published elsewhere.^30,31^

*Cannabis Use.* Respondents were asked if they had ever tried marijuana or hashish and if they had used marijuana or hashish in the past 12 months. Respondents who indicated using marijuana or hashish only 1 time were coded into the ‘No’ group for both lifetime and past 12-month use since one-time users would be different from those using marijuana or hashish more often. The frequency of the past 12-month cannabis use was also assessed but was only examined descriptively due to limited statistical power with this variable.

*Child Maltreatment.* Child maltreatment occurring before 16 years of age included physical abuse, sexual abuse, emotional abuse, neglect, and exposure to intimate partner violence (IPV). Physical abuse was assessed with 3 items and coded as present if the respondent reported being: (a) slapped on the face, head, or ears, or hit or spanked with something hard (3 or more times); (b) pushed, grabbed, or shoved, or having something thrown at the respondent to hurt them (3 or more times); and/or (c) kicked, bit, punched, choked, burned or physically attacked (1 time or more).^32^ Sexual abuse was measured using 2 items and coded as present if the respondent reported: (a) attempted or forced into unwanted sexual activity by being threatened, held down, or hurt in some way (1 time or more) and/or (b) sexually touched, meaning unwanted sexual touching or grabbing, kissing, or fondling (1 time or more). Emotional abuse was assessed using 1 item and coded as present if the respondent reported that a parent or other adult in the home said mean or hurtful things that made the respondent upset or feel really bad about themselves (6 or more times).^32^ Two items were used to assess neglect and included: (a) had to go without things the respondent needed, like food, clothes, shoes, or school supplies (1 time or more) and/or (b) had been left alone or unsupervised before 10 years of age (1 time or more). One item was used to assess exposure to IPV and was coded as present if the respondent ever saw or heard parents, step-parents, or guardians hitting each other or another adult in the home (3 or more times).^32^ A child maltreatment variable (yes or no) was computed to measure experiencing any of the 5 child maltreatment types assessed.

*Deployment-Related Traumatic Events (DRTEs).* Exposure to DRTEs during a CAF deployment was assessed using 10 dichotomous items (yes or no) using the deployment experiences scale.^31^ If a respondent had never been deployed, they were coded as ‘No’ on all of the items. Two items were combined to assess military sexual trauma (i.e., ever sexually assaulted while on a CAF deployment and/or ever experienced any unwanted sexual touching while on a CAF deployment) and 8 items assessed exposure to other types of DRTEs. A dichotomous variable indicating any DRTE (yes or no) was computed based on whether the respondent reported exposure to 1 or more of any of the 10 DRTE items assessed. In addition, a 3-level deployment and DRTE variable were also computed to assess no deployment, deployment without DRTEs, and deployment with DRTEs.

**Table 3.** Child Maltreatment, Deployment-Related Traumatic Experiences, and Cannabis Use Among Canadian Veterans.

	No Past 12-Month Cannabis Use % (95% CI)	Past 12-Month Cannabis Use % (95% CI)	AOR-1 (95% CI)	AOR-2 (95% CI)
**Types of child maltreatment**				
Physical abuse	13.7 (11.6 to 16.3)	20.3 (17.2 to 23.9)	1.70 (1.22 to 2.36)**	1.64 (1.14 to 2.35)**
Sexual abuse	15.2 (13.3 to 17.3)	26.9 (21.1 to 33.7)	2.41 (1.55 to 3 to 73)***	2.31 (1.42 to 3.75)***
Emotional abuse	15.7 (13.7 to 17.9)	19.7 (15.6 to 24.6)	1.31 (0.90 to 1.89)	1.10 (0.72 to 1.66)
Neglect	14.4 (12.3 to 16.8)	20.7 (17.1 to 24.7)	1.53 (1.11 to 2.10)**	1.37 (0.95 to 1.97)
Exposure to IPV	16.3 (14.3 to 18.4)	19.7 (14.3 to 26.4)	1.26 (0.82 to 1.92)	0.88 (0.52 to 1.49)
**Type of DRTE**				
Any deployment-related sexual trauma	16.1 (14.2 to 18.1)	29.8 (19.5 to 42.6)	2.45 (1.25 to 4.79)**	1.57 (0.75 to 3.31)
Known someone who was seriously injured or killed	13.6 (11.4 to 16.2)	19.7 (16.8 to 22.9)	1.42 (1.03 to 1.96)*	1.14 (0.78 to 1.68)
Found yourself in a threatening situation where you were unable to respond because of rules of engagement	15.0 (13.0 to 17.2)	20.6 (16.8 to 25.0)	1.45 (1.03 to 2.04)*	1.01 (0.67 to 1.53)
Ever been injured	13.7 (11.7 to 15.9)	23.4 (19.4 to 27.9)	1.64 (1.18 to 2.30)**	1.39 (0.95 to 2.07)
Ever seen ill or injured women or children who you were unable to help	14.4 (12.4 to 16.7)	20.6 (17.3 to 24.5)	1.49 (1.10 to 2.01)**	1.02 (0.72 to 1.45)
Ever received incoming artillery, rocket or mortar fire	15.2 (13.3 to 17.5)	19.6 (16.1 to 23.7)	1.34 (0.96 to 1.86)	1.01 (0.68 to 1.49)
Ever felt responsible for the death of Canadian or ally personnel	15.8 (13.9 to 17.8)	30.3 (21.3 to 41.1)	1.91 (1.08 to 3.40)*	1.25 (0.62 to 2.54)
Ever had a close call, for example, shot or hit but protective gear saved you	14.9 (13.0 to 17.0)	23.6 (18.7 to 29.3)	1.56 (1.06 to 2.30)*	1.15 (0.70 to 1.88)
Ever had difficulty distinguishing between combatants and noncombatants	15.4 (13.3 to 17.6)	20.9 (16.8 to 25.7)	1.37 (0.94 to 2.00)	1.06 (0.68 to 1.65)

*Note.* CI = confidence interval; AOR = adjusted odds ratio; DRTE = deployment-related traumatic experiences; NS = not statistically significant; IPV = intimate partner violence. AOR-1 models adjust for sex, race, marital status, total household income, education, and last military environment. AOR-2 models adjust for AOR-1 plus past 12-month mental disorders (i.e., posttraumatic stress disorder, major depressive episode, generalized anxiety, panic disorder, social anxiety disorder, alcohol use disorder, and any chronic pain condition).

*p ≤ 0.05; **p ≤ 0.01; ***p ≤ 0.001.

*Mental Disorders.* The World Health Organization version of the Composite International Diagnostic Interview based on DSM-IV diagnostic criteria was used to assess common mental disorders.^33–36^ Past 12-month disorders included generalized anxiety disorder, panic disorder, social phobia, major depressive episode, and alcohol use disorder. An algorithm using several variables was computed to create a past 12-month PTSD diagnosis since a past-year diagnosis was not directly assessed. A past 12-month PTSD diagnosis was assessed using 3 criteria: (a) the presence of a composite international diagnostic interview (CIDI)-based PTSD diagnosis in the 16-year follow-up; (b) responding ‘yes’ to a single question that assessed whether the individual had PTSD-related reactions in the past 12 months; and (c) at least 3 of the 7 PTSD symptoms that were assessed in a past-year time frame.

*Chronic Pain Conditions.* Any chronic pain condition was assessed based on whether the respondent self-reported having a physician or health-care provider diagnose them with any of the following conditions: arthritis, back problems, migraine headaches, and/or any gastrointestinal conditions (i.e., irritable bowel syndrome, inflammatory bowel disease, Crohn's disease, ulcerative colitis, or intestinal or stomach ulcers).

*Sociodemographic and Military Covariates.* Sociodemographic and military covariates included sex (male, female), age (continuous), race/ethnicity (White, non-White), total past-year household income (<$50,000, $50,000 to $99,999, $100,000 to $149,999, $150,000 or more), the highest level of education (less than high school, high school diploma or equivalent, some postsecondary [less than a bachelor's degree], bachelor's degree or higher), and last military environment (air, land, and sea).

### Statistical Analyses

Data were weighted in all analyses to ensure the estimates were representative of the original 2002 CAF study sample. Bootstrapping was the variance estimation technique employed to account for the complex survey design. First, descriptive statistics were computed to examine the prevalence of cannabis use and the distribution of sociodemographic and military covariates, child maltreatment history, and DRTEs by past 12-month cannabis use. Second, logistic regression models were computed to determine if child maltreatment histories and DRTEs were associated with an increased likelihood of past 12-month cannabis use after adjusting, first, for sociodemographic and military variables, adjusted odds ratio-1 (AOR-1) and, additionally, for mental disorders and chronic pain conditions (AOR-2). Sex differences in these relationships were also examined with interaction effects. Third, logistic regression models were computed to determine the interactive and cumulative effects of a child maltreatment history and DRTEs on past 12-month cannabis use. STATA software was used to conduct the statistical analysis.

## Results

Table 1 provides lifetime and past 12-month cannabis use prevalence including sex differences. The prevalence of lifetime and past 12-month cannabis use in the overall sample was 49.4% and 16.7%, respectively. Sex differences were noted for lifetime cannabis use with females being less likely to report compared to males (odds ratio [OR] = 0.71; 95% confidence interval (CI), 0.59 to 0.86). However, no statistically significant sex differences were found for past 12-month cannabis use. Among those who use cannabis in the past 12 months, 43.6% reported using it as much as every day. Table 2 provides the sociodemographic characteristics, military variables, and child maltreatment and DRTE histories stratified by past 12-month cannabis use. Older age, household income of $150,000 or more (compared to <$50,000), and having a bachelor's degree or higher (compared to less than a high school diploma) were all associated with a decreased likelihood of past 12-month cannabis use. Being separated/divorced/widowed (compared to married/common-law), experiencing any child maltreatment and any DRTEs were associated with an increased likelihood of past 12-month cannabis use.

**Table 1.** Prevalence of Cannabis Use Among Canadian Veterans.

Cannabis Use	Total % (95% CI)	Males % (95% CI)	Females % (95% CI)	OR (95% CI)
Lifetime cannabis use				
No	50.6 (48.2 to 53.0)	49.6 (47.0 to 52.3)	58.1 (54.1 to 62.0)	1.00
Yes	49.4 (47.0 to 51.8)	50.4 (47.7 to 53.0)	41.9 (38.0 to 45.9)	0.71 (0.59 to 0.86)***
Past 12-month cannabis use				
No	83.3 (81.3 to 85.2)	83.0 (80.7 to 85.1)	85.9 (82.8 to 88.5)	1.00
Yes	16.7 (14.8 to 18.7)	17.0 (14.9 to 19.3)	14.1 (11.5 to 17.2)	0.80 (0.60 to 1.07)
Frequency of past 12-month cannabis use^a^				
Less than once a month	31.5 (25.6 to 38.1)			
1 to 3 times a month	10.3 (7.0 to 15.0)			
Once a week	3.0 (1.5 to 5.8)			
More than once a week	11.7 (8.0 to 16.7)			
Every day	43.6 (37.1 to 50.3)			

*Note.* CI = confidence interval; OR = odds ratio. As per Statistics Canada data release guidelines, sex-stratified models regarding the frequency of the past 12-month cannabis use were not released to protect participant confidentiality (shaded cells in table).

^a^ Frequency of use only includes participants reporting past 12-month cannabis use.

*p ≤ 0.05; **p ≤ 0.01; ***p ≤ 0.001.

**Table 2.** Association of Sociodemographic and Military Covariates With Past 12-Month Cannabis Use.

Covariate	No Past 12-Month Cannabis Use	Past 12-Month Cannabis Use	OR (95% CI)
	% (95% CI)	% (95% CI)	
Sex			
Male	83.0 (80.7 to 85.1)	17.0 (14.9 to 19.3)	1.00
Female	85.9 (82.8 to 88.5)	14.1 (11.5 to 17.2)	0.80 (0.60 to 1.07)
Age (mean, SE)	54.1 (0.18)	51.0 (0.51)	0.95 (0.93 to 0.97)***
Race/ethnicity			
White	83.2 (81.1 to 85.1)	16.8 (14.9 to 18.9)	1.00
Non-White	87.6 (77.2 to 93.7)	12.4 (6.3 to 22.8)	0.70 (0.32 to 1.52)
Marital status 2018			
Married/common-law	84.5 (82.3 to 86.5)	15.5 (13.5 to 17.7)	1.00
Single, never married	82.5 (72.7 to 89.3)	17.5 (10.7 to 27.3)	1.15 (0.62 to 2.15)
Separated, divorced, widowed	76.6 (69.3 to 82.5)	23.4 (17.5 to 30.7)	1.67 (1.11 to 2.51)*
Household income 2018			
Less than $50,000	77.2 (67.0 to 84.9)	22.8 (15.1 to 33.0)	1.00
$50,000 to $99,999	80.1 (76.1 to 83.6)	19.9 (16.4 to 23.9)	0.84 (0.47 to 1.48)
$100,000 to $149,999	84.9 (80.7 to 88.4)	15.1 (11.6 to 19.3)	0.60 (0.33 to 1.10)
$150,000 or more	86.8 (83.4 to 89.6)	13.2 (10.4 to 16.6)	0.51 (0.28 to 0.94)*
Highest level of education in 2018			
Less than high school	81.3 (69.8 to 89.0)	18.7 (11.0 to 30.2)	1.00
High school diploma or equivalent	81.5 (77.8 to 84.7)	18.5 (15.3 to 22.2)	0.98 (0.48 to 1.99)
Some postsecondary (less than a Bachelor's degree)	81.5 (78.1 to 84.5)	18.5 (15.5 to 21.9)	0.98 (0.49 to 1.97)
Bachelor's degree or higher	92.4 (89.3 to 94.7)	7.6 (5.3 to 10.7)	0.36 (0.16 to 0.78)**
Last military environment			
Air	84.5 (81.2 to 87.2)	15.5 (12.8 to 18.8)	1.00
Land	83.1 (79.7 to 86.1)	16.9 (13.9 to 20.3)	1.10 (0.79 to 1.54)
Sea	81.7 (77.0 to 85.7)	18.3 (14.3 to 23.0)	1.22 (0.84 to 1.75)
Any child maltreatment			
No	88.2 (85.2 to 0.91)	11.8 (9.4 to 14.8)	1.00
Yes	80.2 (77.3 to 82.8)	19.8 (17.2 to 22.7)	1.84 (1.34 to 2.53)***
Any deployment-related trauma			
No	87.5 (84.4 to 90.1)	12.5 (9.9 to 15.6)	1.00
Yes	81.3 (78.6 to 83.8)	18.7 (16.2 to 21.4)	1.62 (1.18 to 2.21)**
Deployment status			
Never deployed	86.4 (81.8 to 90.0)	13.6 (10.0 to 18.2)	1.00
Deployed without DRTE exposure	89.1 (84.4 to 92.6)	10.9 (7.4 to 16.6)	0.77 (0.43 to 1.36)
Deployed with DRTE exposure	81.3 (78.6 to 83.8)	18.7 (16.2 to 21.4)	1.46 (0.99 to 2.15)

*Note.* CI = confidence interval; OR = adjusted odds ratio; SE = standard error; DRTE = deployment-related traumatic event.

*p ≤ 0.05; **p ≤ 0.01; ***p ≤ 0.001.

Table 3 provides findings of the relationship between child maltreatment types and DRTEs with past 12-month cannabis use. Physical abuse, sexual abuse, and neglect were associated with increased odds of past 12-month cannabis use after adjusting for sociodemographic and military variables (AOR-1 ranging from 1.53 to 2.41). Emotional abuse and exposure to IPV were not statistically significant but could reflect a Type II error due to underpowered models. When further adjusting for mental disorders and chronic pain conditions, physical abuse and sexual abuse remained statistically significant (AOR-2 = 1.64 and 2.31, respectively). No statistically significant interaction terms were found for sex and child maltreatment. All DRTEs were associated with past 12-month cannabis use in the models adjusting for sociodemographic and military variables except for ever receiving incoming artillery/rocket fire or ever having had difficulty distinguishing between combatants and noncombatants, possibly due to underpowered models (AOR-1 ranging from 1.42 to 2.45). When further adjusting for mental disorders and chronic pain conditions, all other DRTEs were no longer statistically significant. No statistically significant sex differences were found in these relationships when examining interaction effects.

Table 4 provides the findings for the independent and interactive effects of child maltreatment and DRTEs on the past 12-month cannabis use. In individual models adjusting for sociodemographic and military variables, any child maltreatment (AOR-1 = 1.93; 95% CI, 1.37 to 2.72) and any DRTE (AOR-1 = 1.58; 95% CI, 1.11 to 2.23) were both individually associated with past 12-month cannabis use. When child maltreatment and DRTEs were both put into the same models with the same covariates, both child maltreatment (AOR-1 = 1.86; 95% CI, 1.32 to 2.63) and DRTEs (AOR-1 = 1.50; 95% CI, 1.06 to 2.13) were independently associated with past 12-month cannabis use. When further adjusting for mental disorders and any chronic pain condition, DRTEs became nonsignificant in both the independent models and when entered with any child maltreatment. However, any child maltreatment history remained statistically significant (AOR-2 = 1.88; 95% CI, 1.29 to 2.75) independent of DRTEs, mental disorders, and chronic pain. The interaction effect of any child maltreatment and any DRTEs were nonsignificant in all models.

**Table 4.** Independent and Interactive Effects of CM and DRTEs on Past 12-Month Cannabis Use.

Model	AOR-1 (95% CI)	AOR-2 (95% CI)
Model 1		
Any CM	1.93 (1.37 to 2.72)***	1.92 (1.31 to 2.81)***
Model 2		
Any DRTE	1.58 (1.11 to 2.23)**	1.25 (0.85 to 1.84)
Model 3		
Any CM	1.86 (1.32 to 2.63)***	1.88 (1.28 to 2.75)***
Any DRTE	1.50 (1.06 to 2.13)***	1.20 (0.81 to 1.77)
Model 4		
Any CM*Any DRTE Interaction	0.85 (0.39 to 1.86)	0.93 (0.40 to 2.18)

*Note.* CI = confidence interval; AOR = adjusted odds ratio; CM = child maltreatment; DRTE = deployment-related traumatic event. AOR-1 models adjust for sex, race, marital status, total household income, education, and last military environment. AOR-2 models adjust for AOR-1 plus past 12-month mental disorders (i.e., posttraumatic stress disorder, major depressive episode, generalized anxiety, panic disorder, social anxiety disorder, alcohol use disorder, and any chronic pain condition).

Model 1 enters any CM variable (but not any DRTE variable) along with corresponding AOR-1 or AOR-2 covariates.

Model 2 enters any DRTE variable (but not any CM variable) along with corresponding AOR-1 or AOR-2 covariates.

Model 3 enters any CM and any DRTE variables into the model simultaneously along with corresponding AOR-1 or AOR-2 covariates.

Model 4 enters any CM by any DRTE interaction term into the model along with main effects and corresponding AOR-1 or AOR-2 covariates.

*p ≤ 0.05; **p ≤ 0.01; ***p ≤ 0.001.

Table 5 provides the findings for the cumulative effects of child maltreatment and DRTEs during the past 12-month cannabis use. The 4 groups in the models included: (a) those with no child maltreatment history or DRTEs, (b) those with a child maltreatment history but no DRTEs, (c) those with DRTEs but no child maltreatment history, and (d) those with both child maltreatment history and DRTEs. In models adjusting for sociodemographic and military variables, child maltreatment with (AOR-1 = 2.89; 95% CI, 1.60 to 5.21) and without DRTEs (AOR-1 = 1.97; 95% CI, 1.03 to 3.79) was associated with past 12-month cannabis use. The association with past 12-month cannabis use was greater when experiencing both child maltreatment history and DRTEs together compared to DRTEs alone but was not statistically significantly different from child maltreatment alone. When further adjusting for mental disorders and any chronic pain condition, only child maltreatment and DRTEs were associated with an increased likelihood of past 12-month cannabis use (AOR-2 = 2.20; 95% CI, 1.17 to 4.11).

**Table 5.** Cumulative Effects of CM and DRTEs on Past 12-Month Cannabis Use.

	No CM and No DRTEs	CM Without DRTEs^a^	DRTEs Without CM^b^	CM and DRTE^c^	Significant Differences^1^
AOR-1 (95% CI)	1.00	1.97 (1.03 to 3.79)*	1.62 (0.85 to 3.11)	2.89 (1.60 to 5.21)***	b < c
AOR-2 (95% CI)	1.00	1.84 (0.92 to 3.63)	1.21 (0.59 to 2.46)	2.20 (1.17 to 4.11)*	b < c

*Note.* CI = confidence interval; AOR = adjusted odds ratio; CM = child maltreatment; DRTE = deployment-related traumatic event. AOR-1 models adjust for sex, race, marital status, total household income, education, and last military environment. AOR-2 models adjust for AOR-1 plus past 12-month mental disorders (i.e., posttraumatic stress disorder, major depressive episode, generalized anxiety, panic disorder, social anxiety disorder, and alcohol use disorder) and any chronic pain condition.

^1^ Statistically significant differences at *p* < 0.05 in the prevalence of cannabis use across the different mutually exclusive CM and DRTE categories were tested by changing the reference group in logistic regression models. Letters a, b, and c represent the categories and when were statistically different.

*p ≤ 0.05; **p ≤ 0.01; ***p ≤ 0.001.

## Discussion

The current study found the overall prevalence of lifetime cannabis use among veterans to be lower among females compared to males, consistent with previous research from the US.^9,22^ However, differences were not noted in the past 12-month cannabis use between male and female Canadian veterans. Any work in this area should include male and female veterans.

The most robust association with the past 12-month cannabis use was the experience of childhood physical abuse and sexual abuse, independent of sociodemographic characteristics, military covariates, mental disorders, chronic pain conditions, and DRTEs. Any child maltreatment also remained independently associated with past 12-month cannabis use. The effect sizes of the relationship between child maltreatment and cannabis use in this veteran sample were similar in size to those found in general population samples^37–39^. Although, due to differences in the data, direct comparisons cannot be made. Importantly, the findings from the current study indicate how child maltreatment can continue to have impacts across the lifespan and also extends existing evidence from other studies that indicate that child maltreatment is associated with an increased likelihood of suicide-related behaviour and mental disorders in military samples.^19,20^ Accordingly, prevention efforts aimed at reducing child maltreatment are of utmost importance, alongside evidence-based interventions that may reduce the onset of cannabis use. Persons working with military and veteran populations should understand the link between child maltreatment and cannabis use. Importantly, it should be noted that even though robust relationships were found between trauma and cannabis use, a large proportion of veterans using cannabis did not experience child maltreatment or DRTEs. This highlights the need to understand other reasons for cannabis use in this population.

Experiencing trauma while in the military is also an important factor in understanding cannabis use. Notably, similar proportions of past 12-month cannabis use were found for those who never deployed and those who deployed without DRTEs, but a higher proportion for those who deployed with DRTEs although this OR did not quite reach statistical significance. Furthermore, almost all DRTEs were associated with the past 12-month cannabis use when adjusting for sociodemographic and military covariates. However, when accounting for mental disorders and any chronic pain condition, all findings were attenuated and became nonsignificant. This may indicate that the variance in the relationship between DRTEs and the past 12-month cannabis use is accounted for by mental disorders and chronic pain conditions. The present results align with a self-medication hypothesis: vulnerability to distress may arise from early or later traumas, subsequently manifest in diagnosable mental health disorders, which are subsequently self-medicated with cannabis (or other drugs).^40^ Importantly this study was not able to assess if veterans were using cannabis specifically as a means of coping with mental health or physical health problems such as pain. More research in the area is warranted.

When examining cumulative effects, child maltreatment alone without DRTEs was significantly associated with increased odds of cannabis use, whereas DRTEs without child maltreatment were not. However, cumulative effects were found, which indicated that having experienced both child maltreatment and DRTEs was linked with a greater likelihood of past 12-month cannabis use, compared to DRTEs without child maltreatment, but not statistically significantly different from child maltreatment only. Clinically, if treatment is needed for cannabis use, then child maltreatment, DRTEs, mental disorders, and pain conditions may all need to be understood to inform effective treatment strategies that address the complex interplay, either sequentially or concurrently.^41^ In any case, the recent use of cannabis as a PTSD treatment may create complications with respect to assessment and treatment.^42^

Limitations of the current research should be noted. First, child maltreatment was retrospectively assessed in adulthood, which may result in recall bias. However, there is evidence that shows retrospective recall in survey data to assess trauma in childhood is valid and reliable.^43–45^ Second, because the data were cross-sectional, inferences regarding causation cannot be made. However, assessing the past 12-month cannabis use helps to establish a temporality of exposure to outcome. Third, the current study assessed the use of marijuana and hashish in early 2018, which was several months before cannabis became legal and more widely accessible in Canada. Thus, the current findings may represent conservative estimates of cannabis use since disclosure may have been less likely before legislative change. Additionally, this study was not able to distinguish between medical and recreational cannabis use or to assess those who would meet the criteria for cannabis use disorder. Entirely disentangling medical and recreational cannabis use would be difficult because both could be related to coping. The frequency of cannabis use could not be examined in models due to limited statistical power. Fourth, these data only allowed assessment of conditions associated with chronic pain, not chronic pain itself. Finally, sex differences for cumulative and interaction models could not be computed due to insufficient power.

The current findings highlight the importance of a comprehensive understanding of trauma histories, including child maltreatment and DRTEs, and the impact these experiences may have on cannabis use and veteran health across the lifespan. Further work is needed to understand the role cannabis use may play related to self-medication or as a means of coping among veterans. From a public health perspective, the prevention of child maltreatment remains a priority and may, over time, lead to better health in the overall population including among veterans.
